# First Australian experience of treating localised prostate cancer patients with CyberKnife stereotactic radiotherapy: early PSA response, acute toxicity and quality of life

**DOI:** 10.1002/jmrs.205

**Published:** 2017-03-08

**Authors:** Ashutosh Dixit, Colin Tang, Sean Bydder, Mary‐Anne Kedda, Eva Vosikova, Chrianna Bharat, Suki Gill

**Affiliations:** ^1^ Department of Radiation Oncology Sir Charles Gairdner Hospital Nedlands Western Australia Australia; ^2^ Department of Surgery University of Western Australia Crawley Western Australia Australia; ^3^ School of Population Health University of Western Australia Crawley Western Australia Australia; ^4^ Centre for Applied Statistics University of Western Australia Crawley Western Australia Australia; ^5^ Department of Research Sir Charles Gairdner Hospital Nedlands Western Australia Australia

**Keywords:** CyberKnife, prostate cancer, quality of life, stereotactic radiotherapy

## Abstract

**Introduction:**

This study is to evaluate biochemical response, acute toxicity and health‐related quality‐of‐life (QOL) outcomes among prostate cancer patients following stereotactic body radiation therapy (SBRT) in the first Australian CyberKnife facility.

**Methods:**

Forty‐five consecutive patients with clinically localised prostate cancer were treated with SBRT using CyberKnife technology and enrolled in this study. Protocol treatment consisted of 36.25 Gy in five fractions. PSA and acute toxicity was assessed at each follow‐up visit and QOL was assessed using the European Organisation for Research and Treatment of Cancer (EORTC) Global Health Status (GHS) C30 and PR25 questionnaires and the Karnofsky Performance Status (KPS). Distance of travel for treatment was recorded.

**Results:**

The median prostate‐specific antigen (PSA) level declined from the initial value of 6.9 ng/mL to 1.5 ng/mL at 6 months and 0.6 ng/mL at 18 months post‐treatment. Results were similar in patients who did not receive hormone therapy. Acute grade 1 gastrointestinal (GI) and genitourinary (GU) toxicities were found in 11.1% and 24.4% of patients respectively. Acute grade 2 GI and GU toxicities were found in 2.2% and 11.1% of patients respectively. There were no grade 3 and grade 4 toxicities. Mean urinary symptom score was 14.8 at baseline, 17.2 at 6 weeks and 18.3 at 6 months (*P* > 0.05). Mean bowel symptom score was 2.7 at baseline, 4.2 at 6 weeks and 6.3 at 6 months (*P* > 0.05). The mean GHS score improved from 81.3 at baseline to 82.4 at 6 weeks, and was 75.6 at 6 months (*P* > 0.05, not significant). Compared to baseline KPS, there was a significant mean decrease from baseline of 96.7 to 93.3 at the 6‐week follow‐up (*P* = 0.0043), which then recovered to 94.3 at the 6‐month follow‐up (*P* = 0.1387).

**Conclusions:**

Early results show promising PSA response. Acute toxicity seemed comparable to results from conventionally fractionated radiotherapy and to international prostate SBRT studies. EORTC PR25 and C30 scores did not reveal any significant change from baseline, and although there was a decrease in KPS, the absolute decrease was small.

## Introduction

Stereotactic body radiation therapy (SBRT) is a relatively new treatment option for clinically localised prostate cancer whereby radical treatment is complete in five fractions.^1^

There remains some uncertainty regarding long‐term outcomes compared to conventional treatment, but the treatment is being increasingly used worldwide. Although initial stereotactic prostate radiotherapy studies used a standard linear accelerator,^2^ CyberKnife robotic radiosurgery is now more commonly used because of its ability to track the prostate. CyberKnife robotic radiosurgery uses a linear accelerator mounted on a robotic arm. It uses hundreds of beams that can be delivered non‐isocentrically. The systems used kilovoltage imaging to check target positioning and will automatically correct displacement in real‐time for all six degrees of motions. Using continuous tracking of gold fiducial markers inserted into the prostate, planning target volume (PTV) expansion margins with CyberKnife are typically 2–3 mm posterior and 5 mm in all other directions, and with these expansion margins rectal toxicity appears tolerable.^1^

As prostate SBRT is a relatively new option for prostate cancer, only limited reports of its impact on quality of life (QOL) are available. At present our institute is the only one in Australia with a CyberKnife facility, and we started using CyberKnife SBRT for prostate cancer treatment in 2014. We have prospectively collected prostate‐specific antigen (PSA), toxicity and QOL data in every patient who agreed to participate in the data collection for the first year. This is the first report of that data.

## Methods and Materials

This study was approved by the Sir Charles Gairdner group human research ethics committee (2014‐031) and participants provided written consent for their data to be used. Data were collected for patients treated between April 2014 and April 2015 prospectively with planned data collection points at 6 weeks and 6, 12 and 18 months. Data collection close‐out date was 30 September 2016. Every patient treated with CyberKnife at our centre is also invited to enrol in the International RSSearch^®^ Patient Registry (ClinicalTrials.gov Identifier: NCT01885299), and as such some of the data presented in this study have been reported in part as a pooled analysis with other centres.^3^ All patients had clinically localised biopsy‐proven prostate cancer, confirmed by computed tomography (CT) and bone scan. Patients were stratified into D'Amico risk groups (low risk: PSA < 10, Gleason sum of 6 and clinical stage T1c–T2a; intermediate risk: PSA 10–20, Gleason sum of 7 or clinical stage T2b; high risk: PSA > 20, Gleason sum of 8–10 or clinical stage T2c or higher). Short‐term (3–6 months) androgen deprivation therapy (ADT) was prescribed at the discretion of the treating radiation oncologist.

### Treatment planning and delivery

**Figure 1:**
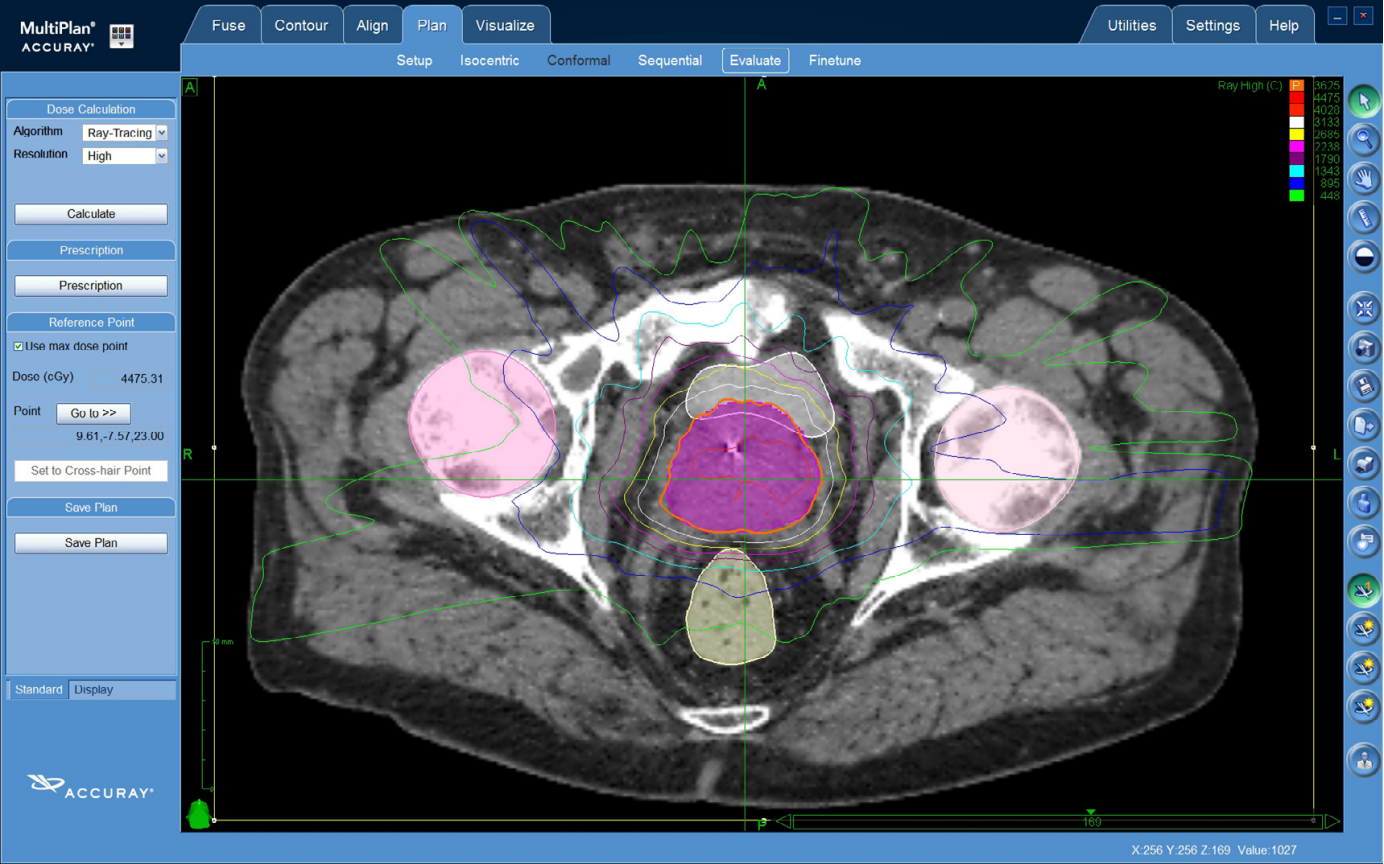
A sample CyberKnife SBRT plan.

Patients were given instructions for bladder and bowel preparation to be used before the planning CT scan and before every fraction. Patients were advised to take one sachet of Movicol the evening before each fraction, take simethicone three times a day, drink 1.5 L water a day and to empty their bladder and drink 250 mL of water 30 min prior to treatment. A planning CT and magnetic resonance imaging (MRI) scan was conducted approximately 1 week after the ultrasound guided insertion of four gold fiducial markers into the prostate for image guidance. CT was conducted with 1‐mm thick slices 15 cm superior and inferior to the centre of fiducials. Two sequences of MRI prostate (T1 and T2) were fused to the planning CT; the T1 sequences were used for gold fiducial fusion and T2 sequences were used for delineation of the target. Membranous urethra was contoured based on T2 MRI, commencing from the slice inferior to the apex of the prostate. Dose was prescribed to the planning target volume (PTV), which consisted of a 5‐mm expansion on the clinical target volume (CTV) in all directions except posterior where a 2‐ to 3‐mm expansion was used. CTV included the entire prostate, all visible extension of tumour outside the prostate and entire seminal vesicles (SV) if there is SV invasion (stage T3b) and only proximal SV if risk of invasion is >15% as per Partin's table. Homogeneous non‐isocentric planning was performed using Multiplan^™^ (Accuray, Inc., Sunnyvale, CA, USA), and dose normalised to the 70–80% isodose line so that the prescription isodose covered >95% of the PTV. Typical dose targets and constraints for critical organs are shown in Table 1. SBRT was delivered using the CyberKnife system, M6 FIM model. A sample CyberKnife SBRT plan is shown in Figure 1. During a typical 45‐min treatment, fiducial seeds were tracked and target position was verified at 15–45 sec intervals. Displacement is corrected automatically. Our prescription protocol was 36.25 Gy in five fractions on alternate days, but two patients were prescribed a lower dose. One received 35 Gy in five fractions because he had prior radiotherapy to the pelvis for rectal cancer in 1997. A second patient received 32.4 Gy in five fractions because he had a prosthetic right hip and a large prostate, and the prescription dose was dropped to meet dose constraints.

**Table 1:** Typical dose targets and constraints

Global max dose	≤48.33 Gy
PTV	V36.25 Gy ≥ 95%
Rectum	V (36 Gy) < 1 cc
	V (36.25 Gy) < 5%
	V (32.62 Gy) < 10%
	V (29 Gy) < 20%
	V (27.19 Gy) < 25%
	V (18.12 Gy) < 50%
Bladder	V (37 Gy) < 5 cc
	V (36.25 Gy) < 10%
	V (18.12 Gy) < 40%
Membranous urethra	Max < 40 Gy
Bowel	V30 Gy < 1 cc

### Data collection

Data were collected before treatment as baseline, at first follow‐up around 6 weeks post‐treatment and at second follow‐up around 6 months post‐treatment. We also have PSA results done at 12 and 18 months’ follow‐up. The common terminology criteria for adverse events (CTCAE) version 3.0 grading system were used to assess toxicity. A high CTCAE score represents a high level of symptomatology. The highest toxicity score for each patient was used to calculate the CTCAE value. QOL was assessed using European Organisation for Research and Treatment of Cancer (EORTC) C30 and PR25 questionnaires. A high score for global health status (GHS) represents high QOL in the EORTC C30 questionnaire. Karnofsky Performance Status (KPS) was also collected.

### Statistical methods

All QOL scores were calculated using test‐specific guidelines as detailed in the EORTC manuals.^4^ Analyses for QOL were restricted to baseline, 6 weeks and 6 months visits. In the case of missing 6‐month post‐treatment scores, last observation carried forward (LOCF) method was applied by replacing the missing score with the 6‐week score.

Univariate and multivariate linear mixed models were used to compare the change in GHS, urinary symptoms, bowel symptoms and KPS scores from baseline to follow‐up visits after adjusting for age, Gleason score, tumour stage, baseline PSA, D'Amico risk group, ADT and dose. Patients without a baseline measure for the response variable were excluded from that analysis. Transformations were conducted to satisfy model assumptions where necessary. Visit number and the variables adjusted for were considered as a fixed effect and patient was included as a random effect. Variables that were significant at the 5% level were retained for the final model. Visit number remained in the models regardless of its significance as it was the variable of interest. Data were analysed using the R environment for statistical computing.^5^

## Results

Out of 53 patients who underwent prostate SBRT, 45 patients completed their QOL questionnaires and consented to the use of their follow‐up data for this study. Patient characteristics are summarised in Table 2. As per D'Amico risk stratification, 11 patients were low risk, 28 were intermediate risk and 6 were high risk. Of the six high‐risk patients, two patients were T2c, one patient was T3a, Gleason 9 and baseline PSA of 23, one patient was T4 (with early invasion of bladder and levator ani), one patient had Gleason 8 disease and one patient had a baseline PSA of 22. The median age of patients was 71 years (range: 46–86 years). The median PSA pre‐treatment was 6.9 ng/mL (range: 1.69–23.16). Seven patients received ADT, of these three were high risk and four were intermediate risk.

**Table 2:** Patient characteristics

Age at diagnosis	Years
Mean	69.2
Median	71
Range	46–86
PSA level pre‐treatment	ng/mL
Mean	8.2
Median	6.9
Range	1.69–23.16
Risk category	*n* (%)
Low	11 (24.4)
Intermediate	28 (62.3)
High	6 (13.3)
T stage	*n* (%)
T1	22 (49)
T2a	13 (29)
T2b	6 (13)
T2c	2 (4)
T3a	1 (2)
T4	1 (2)
Gleason score	*n* (%)
6	16 (36)
7 (3 + 4)	20 (44)
7 (4 + 3)	7 (16)
8	1 (2)
9 (4 + 5)	1 (2)
Hormone treatment	*n* (%)
Yes	7 (16)
No	38 (84)
Patient's residence	*n*
Regional (<200 km)	29 (64)
Remote (>200 km)	16 (36)

One patient received CyberKnife as he had radiotherapy to the pelvis previously, and margins could be reduced with CyberKnife tracking. Two patients had hip replacements and a better plan was possible with non‐coplanar treatment. The remainder were offered CyberKnife because of patient preference usually for shorter treatment. Sixteen patients (36%) lived more than 200 km away from our treatment centre.

### PSA response

**Figure 2:**
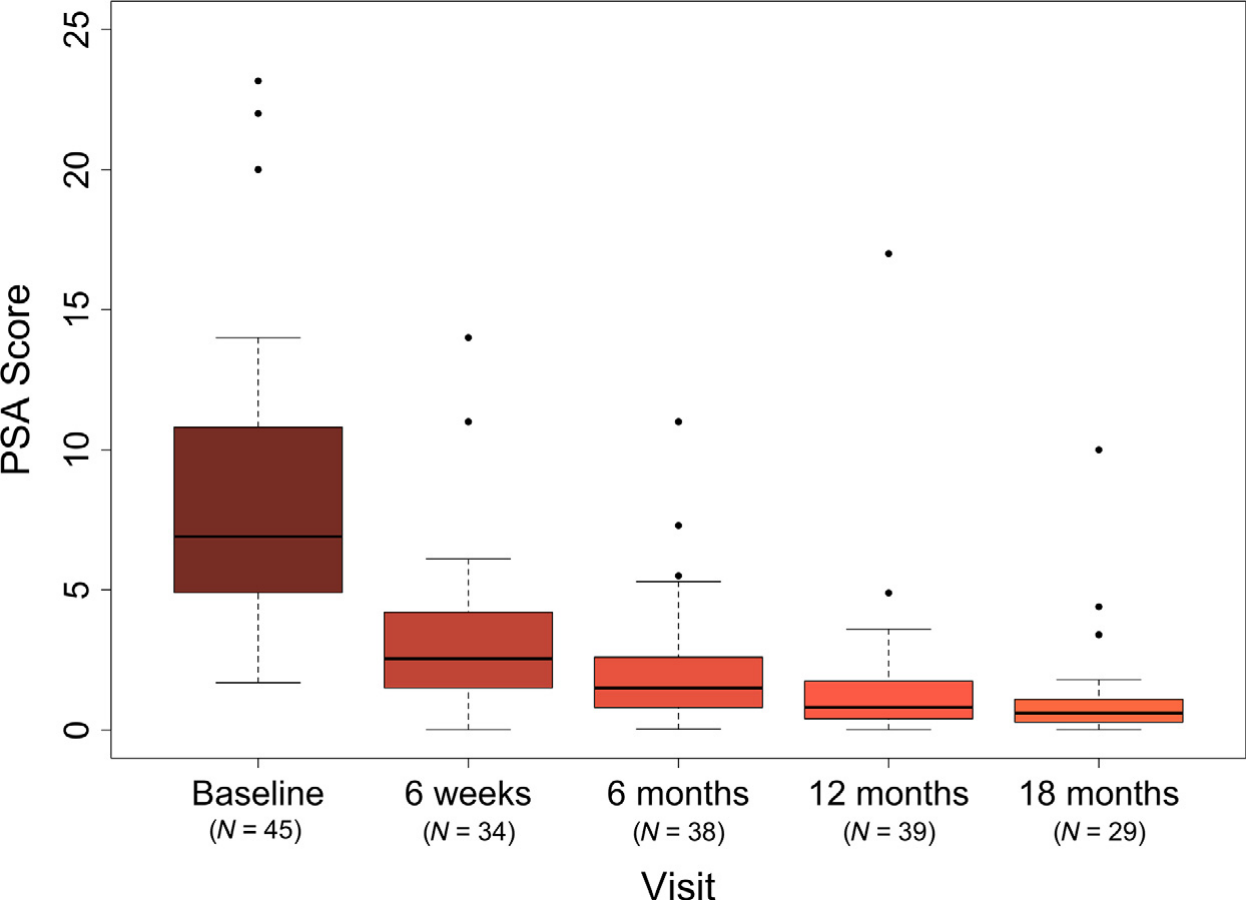
Box plot for PSA response.

Figure 2 shows the box plots for PSA change after treatment. The median PSA level declined from the initial value of 6.9 ng/mL to 1.5 ng/mL at 6 months and 0.6 ng/mL at 18 months post‐treatment. Results were similar in group of patients who did not receive hormone therapy (Table 3). This study showed an excellent median PSA response after SBRT with 78.3% decline over 6 months relative to baseline value. Only one patient did not have early PSA response, with a baseline PSA of 9.7 ng/mL (Stage T1c, Gleason sum 6), which increased to 14 ng/mL at 6 weeks post‐treatment before dropping down to 11 ng/mL at 6 months and 5.5 ng/mL at 10 months.

**Table 3:** PSA response

	Baseline	6 weeks	6 months	12 months	18 months
Whole group					
*n*	45	34	38	39	29
Median PSA (ng/mL)	6.9	2.5	1.5	0.8	0.6
No ADT group					
*n*	38	30	31	34	26
Median PSA (ng/mL)	6.8	2.5	1.6	1.1	0.6

PSA, prostate‐specific antigen; ADT, androgen deprivation therapy.

### Acute toxicity

Acute toxicity data for this study and other studies are summarised in Table 4. Acute grade 1 gastrointestinal (GI) and genitourinary (GU) toxicities were found in 11.1% and 24.4% of patients respectively. Acute grade 2 GI and GU toxicities were found in 2.2% and 11.1% of patients respectively. There were no grade 3 and grade 4 toxicities.

**Table 4:** Acute toxicity data comparison with other studies

Study	*n*	Dose/fractions	Toxicity scale	GI toxicity (%)				GU toxicity (%)			
				G1	G2	G3	G4	G1	G2	G3	G4
SBRT											
This study	45	35–36.25 Gy/5#	CTCAE v3	11.1	2.2	0	0	24.4	11.1	0	0
Chen et al.^7^	100	35–36.25 Gy/5#	CTCAE v3	35	5	0	0	36	35	1	0
Madsen et al.^8^	40	33.5 Gy/5#	RTOG	26	13	0	0	28	20.5	1	0
Jabbari et al.^9^	38	38 Gy/4# or 19 Gy/2# boost after EBRT	CTCAE v3	21	11	0	0	29	42	0	0
Katz et al.^10^	515	35–36.25 Gy/5#	RTOG	77	4	0	0	73	4	0	0
IMRT											
Zelefsky et al.^11^	772	81 Gy/45# or 86.4 Gy/48#	RTOG	22	4	0	0	38	28	0	0
IGRT											
Gill et al.^12^	249	78 Gy/39#	CTCAE v3	49	9	0	0	30	54	9	0

CTCAE, common terminology criteria for adverse events; RTOG, radiation therapy oncology group; GI, gastrointestinal; GU, genitourinary.

### Quality of life

Summary statistics for QOL scales of interest by visit are shown in Table 5 and the change in the scores from baseline to 6 weeks and baseline to 6 months are shown in Table 6. There were no statistically significant changes from baseline to 6 weeks or baseline to 6 months for GHS, urinary or bowel symptom scores (all *P*s > 0.05). The mean GHS score improved from 81.3 at baseline to 82.4 at 6 weeks, and dropped to 75.6 at 6 months. While a high score for GHS represents a high QOL, a high score for urinary and bowel scales represents a high level of symptomatology/problems. Considering this, the mean urinary symptom score was 14.8 at baseline, and gradually increased to 17.2 at 6 weeks and 18.3 at 6 months. Mean bowel symptom score was 2.7 at baseline, which increased to 4.2 at 6 weeks and 6.25 at 6 months.

**Table 5:** Summary statistics for QOL scales by visit

Visit	QOL scale	*N*	Mean	Standard deviation	Min.	Median	Max.
Baseline	GHS	44	81.3	19.9	33.3	87.5	100.0
	Urinary	43	14.8	13.8	0.0	12.5	54.2
	Bowel	43	2.7	6.0	0.0	0.0	25.0
	KPS scale	42	97.0	7.0	70.0	100.0	100.0
6 weeks	GHS	37	82.4	18.3	33.3	83.3	100.0
	Urinary	38	17.2	16.1	0.0	14.6	54.2
	Bowel	38	4.2	10.0	0.0	0.0	41.7
	KPS scale	45	93.0	9.0	70.0	100.0	100.0
6 months	GHS	39	75.6	25.5	16.7	83.3	100.0
	Urinary	38	18.3	20.1	0.0	12.5	75.0
	Bowel	36	6.3	12.7	0.0	0.0	58.3
	KPS scale	35	95.0	7.0	80.0	100.0	100.0
12 months	GHS	7	88.1	13.5	66.7	91.7	100.0
	Urinary	7	11.9	13.3	0.0	4.2	37.5
	Bowel	7	1.2	3.2	0.0	0.0	8.3
	KPS scale	6	98.0	4.0	90.0	100.0	100.0

GHS, Global Health Status; KPS, Karnofsky Performance Status.

**Table 6:** Summary statistics for change in QOL scores from baseline (BL) to 6 weeks and baseline to 6 months

Response	Comparison	*N*	Mean	Standard deviation	Min.	Median	Max.	*P*‐value
QLQ‐C30								
Global Health Status	BL to 6 weeks	33	2.02	16.28	−25.00	0.00	58.33	0.77
	BL to 6 months	34	−3.92	21.93	−66.67	0.00	50.00	0.26
QLQ PR25								
Urinary symptoms	BL to 6 weeks	34	1.12	13.69	−33.33	0.00	41.67	0.46
	BL to 6 months	33	1.82	15.29	−33.33	0.00	45.83	0.86
Bowel symptoms	BL to 6 weeks	34	0.98	7.04	−16.67	0.00	25.00	0.97
	BL to 6 months	32	3.13	11.74	−8.33	0.00	58.33	0.07
Karnofsky score	BL to 6 weeks	41	−0.03	0.09	−0.20	0.00	0.20	0.006
	BL to 6 months	32	−0.02	0.09	−0.20	0.00	0.20	0.13

Mean Karnofsky Performance Status dropped from 96.7 to 93.3 at 6 weeks (*P* = 0.0064), but then recovered to 94.3 at 6 months (*P* = 0.1387 compared to baseline).

## Discussion

CyberKnife SBRT has opened a new avenue of treatment for prostate cancer patients in Australia. We found in this study that the great majority of patients with localised prostate cancer who were treated with CyberKnife had a good initial PSA response with rapid decline by 6 months. We found that acute toxicity was tolerable and that no patient in our cohort experienced a grade 3 or grade 4 toxicity. PR25 symptom scores for bladder and rectal toxicity did not change significantly after treatment. GHS score was also stable compared to baseline.

The early PSA response in our study does agree with other published studies. King et al. recently pooled data from 1100 patients treated with CyberKnife with 36.25 Gy in five fractions.^6^ After a median follow‐up of 36 months, the biochemical relapse‐free survival rate was 95%, 84% and 81% for low‐, intermediate‐ and high‐risk patients respectively. For 135 patients possessing a minimum of 5 years follow‐up, the 5‐year biochemical relapse‐free survival rate for low‐ and intermediate‐risk patients was 99% and 93% respectively. It is reassuring that all but one patient in our cohort had significant drops in their PSAs.

For visual comparison, acute toxicities from this study were tabulated against other SBRT,^7^, ^8^, ^9^, ^10^ intensity modulated radiation therapy (IMRT)^11^ and image‐guided radiation therapy (IGRT)^12^ studies (Table 4). There are studies which suggest that SBRT has an acceptable acute toxicity profile,^7^, ^8^, ^9^, ^10^ but a recent publication by Yu et al. suggests otherwise.^13^ Yu et al. compared costs in patients above the age of 66 who were treated with SBRT and IMRT, and looked at the United State's Medicare claims after treatment as a surrogate measure of toxicity. Treatment cost was much lower for SBRT (mean cost $13,645 for SBRT vs. $21,023 for IMRT), at 24 months after treatment initiation, 43.9% of SBRT versus 36.3% of IMRT patients experienced GU toxicity (odds ratio (OR), 1.38; 95% confidence interval (CI), 1.12–1.63). The design of that study was heavily criticised,^14^ but it does echo anecdotal concerns that in SBRT prostate the urethra is within the target, and that toxicity from hypofractionation may manifest later.^15^ The long‐term data from SBRT are still accumulating, however it is reassuring that patients in our study did not experience significant increased acute toxicity as self‐assessed by the PR25 QOL form.

As a more comprehensive measure of patient well‐being (than toxicity scoring), the EORTC Q30 GHS measure of QOL is a multi‐dimensional concept that includes domains related to physical, mental, emotional and social functioning. Many studies have examined the importance of health‐related QOL after definitive treatment for prostate cancer with brachytherapy and conventionally fractionated radiotherapy.^16^, ^17^ Another study which looked at expanded prostate cancer index composite (EPIC)‐based QOL measures in SBRT prostate patients found a decline in urinary and bowel scores at 2 months post‐treatment, and this returned to baseline at approximately 6 months.^10^ Our study confirms that patients treated with SBRT did not have a decline in the GU, GI or general QOL. However, KPS did decline slightly after SBRT prostate, which is not surprising as lethargy is one of the commonest acute side effects of prostate radiation treatment.^12^ The decline, however, was very small and probably not clinically significant.

There is still some uncertainty about the long‐term results of prostate SBRT. All patients are offered conventional IMRT also. We have found that treatment course duration was important to patients, especially if they lived far from the cancer centre. Although our department does provide free lodging for country patients, 36% of patients who requested SBRT in our study group were country patients. CyberKnife has opened a new avenue of treatment for prostate cancer patients. Distance of travel for radiotherapy was a common reason for selecting CyberKnife over fractionated radiotherapy in this study. Based on published studies, our department usually reserves CyberKnife for low‐ and intermediate‐risk prostate cancer only, but several country patients with high‐risk disease in our study group had previously refused conventionally fractionated radiotherapy because of time of stay in the city, and therefore, were treated with CyberKnife in this study.

The strengths of this study are that it is from a single institution, and data were collected prospectively by a dedicated research team, and that data were anonymised, which may reduce reporting bias. There are several weaknesses of our study. Of 53 CyberKnife prostate patients approached, only 45 initially enrolled in study. Of these 45, only 39 completed the questionnaires at 6 months. Second, while follow‐up was recommended to occur at the defined time points, patients were actually seen at their first post‐treatment follow‐up at a minimum 5 weeks post‐treatment, maximum 20 weeks and the median follow‐up was 12 weeks post‐treatment. It is not certain why some patients were seen much later than the recommended follow‐up times, but patient preference may have been a cause, especially as some patients lived very far away from the cancer centre. However, acute toxicity is defined as toxicity within the first 90 days of treatment, because of the small number of patients treated with CyberKnife at our centre so far, we are confident we managed to capture a representative picture of actual acute toxicity, even in the patients who did not complete the QOL questionnaires. Third, the short follow‐up of this study is a weakness in that PSA reporting may not be reflective of cure, especially if androgen deprivation therapy was used upfront. However, only seven patients in our study received androgen deprivation therapy. We hope to continue with data collection so that late toxicity and biochemical failure will be available in the future. We hope to investigate patient preferences for SBRT, but trading off potential risks versus benefits would require a more formal and structured evaluation than we have done here.^18^ There is great interest in SBRT and there are a number of randomised trials in progress. These include the PACE trial which is a trial comparing surgery, conventional radiotherapy and stereotactic radiotherapy for localised prostate cancer and an Australian TROG SPARK study which is studying stereotactic prostate adaptive radiotherapy utilising KIM (kilovoltage intra‐fraction monitoring).

## Conclusion

Early results from the first CyberKnife treatment facility in Australia show promising PSA response after CyberKnife SBRT for clinically localised prostate cancer. The rates and severity of acute toxicity following CyberKnife SBRT are found to be comparable to patients treated with SBRT in other centres and with other radiation modalities. There was no significant negative effect on the quality of life of patients in our study.

## Conflict of Interest

The authors declare no conflict of interest.
